# Sparse distributed memory: understanding the speed and robustness of expert memory

**DOI:** 10.3389/fnhum.2014.00222

**Published:** 2014-04-28

**Authors:** Marcelo S. Brogliato, Daniel M. Chada, Alexandre Linhares

**Affiliations:** ^1^Behavioral and Decision Sciences, EBAPE/Fundação Getulio VargasRio de Janeiro, Brazil; ^2^Computational Cognitive Science Lab., Department of Psychology, University of CaliforniaBerkeley, CA, USA

**Keywords:** sparse distributed memory, non-linearity, critical distance, theoretical neuroscience, expert memory

## Abstract

How can experts, sometimes in exacting detail, *almost immediately and very precisely* recall memory items from a vast repertoire? The problem in which we will be interested concerns models of theoretical neuroscience that could explain the speed and robustness of an expert's recollection. The approach is based on Sparse Distributed Memory, which has been shown to be plausible, both in a neuroscientific and in a psychological manner, in a number of ways. A crucial characteristic concerns the limits of human recollection, the “tip-of-tongue” memory event—which is found at a non-linearity in the model. We expand the theoretical framework, deriving an optimization formula to solve this non-linearity. Numerical results demonstrate how the higher frequency of rehearsal, through work or study, immediately increases the robustness and speed associated with expert memory.

## 1. Introduction

Szilard told Einstein about the Columbia secondary-neutron experiments and his calculations toward a chain reaction in uranium and graphite. Long afterward [Szilard] would recall his surprise that Einstein had not yet heard of the possibility of a chain reaction. When he mentioned it Einstein interjected, “Daranhabe ich gar nicht gedacht!”-“I never thought of that!” He was nevertheless, says Szilard, “very quick to see the implications and perfectly willing to do anything that needed to be done.”

—July 16, 1941, meeting between Leo Szilard and Albert Einstein concerning atomic weapons (Rhodes, 2012, p. 305).

How can experts—like Albert Einstein—immediately find meaning given very few cues? How can experts—like Leo Szilard—recollect, sometimes in exacting detail, memories that non-experts would find baffling? These abilities span wide across the spectrum of human activity: From full chess games played decades ago, to verses written by Dante, to exotic wines, or to the script and actors involved in movie scenes, experts can *almost immediately and very precisely* recall from a vast repertoire. How can neuroscience explain the speed and robustness of experts' recollection?

The work done herein can be related to the work done by Shepard (1957) and further developed by Nosofsky (1986); Shepard (1987) in the sense that the models investigated here use conceptual approximation and distancing in what could be considered a psychological space. However, this work does not aim to continue these authors' approaches to identification, categorization, similarity and psychological distance. Here we aim at discovering the bounds and limits of conceptual retrieval in human memory via the Sparse Distributed Memory (SDM) proxy.

Recently, Abbott et al. (2013) explored a computational level (as defined by Marr, 1892) account of SDM as a model of inference. We provide here an initial exploration that may further the work done by these authors, providing a theoretical foundation for a computational account of the edges of recollection via Sparse Distributed Memory (and possibly other architectures, by means of the connectionist common-ground).

Other approaches that are neurally plausible could include the template and chunk theory by Gobet et al. (Gobet and Simon, 2000; Gobet et al., 2001; Harré et al., 2012; Harré, 2013). Chunks are stored memory items, and templates include slots in which items can vary.

Recent findings by Huth et al. (2012) suggest that human semantic representation resides in a continuous psychological space. The authors provide evidence in the form of fMRI results supporting that human semantic representation resides in a continuous multidimensional space. The SDM model explored herein is consistent with these findings in that SDM permits hierarchical relationships between concepts, and instantiates a multidimensional conceptual space which holds attractors to memory items that are, in fact, continuous (as a function of their distance from the reading point).

Two of the concepts with which we will deal here are reflected in this 1941 meeting: *information content*, shown by Einstein's surprise involved in unexpected information; and *the ability to rapidly access memory, in detail*, shown by Szilard's “long afterward” recollection of the meeting. A third concept we will use is that evidence points toward memory being organized around cell assemblies, and Sparse Distributed Memory takes advantage of this concept.

## 2. Cell assemblies and the sparse distributed memory model

### 2.1. Cell assemblies

How is information encoded in the brain? We postulate that information is encoded by cell assemblies, not by individual neurons (Sakurai, 1996, 1998, 1999). There are at least five reasons leading to this position. (1) Neurons constantly die—yet the brain is robust to their loss. (2) There is large variability in the activity of individual neurons—as would be expected on anatomical and physiological grounds alone. (3) A single neuron does not participate in a single function; as Sakurai (1998) puts it:

Even the famous “face neurons” in the temporal cortex do not respond to single unique faces but to several faces or to several features comprising the several faces. (p. 2)

(4) Studies of activity correlation between neighboring neurons show very low, if not zero, correlation. (5) Finally, while the number of neurons is quite large, it is minute in comparison with the different combinations of incoming stimuli one experiences during one's lifetime.

Furthermore, recent literature suggests the connection between the increased activation of the fusiform face area (FFA) and the acquisition of expertise (Gauthier et al., 1999; Xu, 2005; McGugin et al., 2012). Current results hold strong evidence that FFA activation is correlated with domain-specific expertise in naturalistic settings (Bilalić et al., 2011b). Additionally, it is shown that expertise in object-recognition tasks modulates activation in different areas of the brain (Bilalić et al., 2011a), including homologous right-left hemispheric activation in both object and pattern recognition expertise (Bilalić et al., 2010, 2012). This evidence and the preceding points serve to further emphasize the distributed role of activation in recognition and expertise.

**Figure 1:**
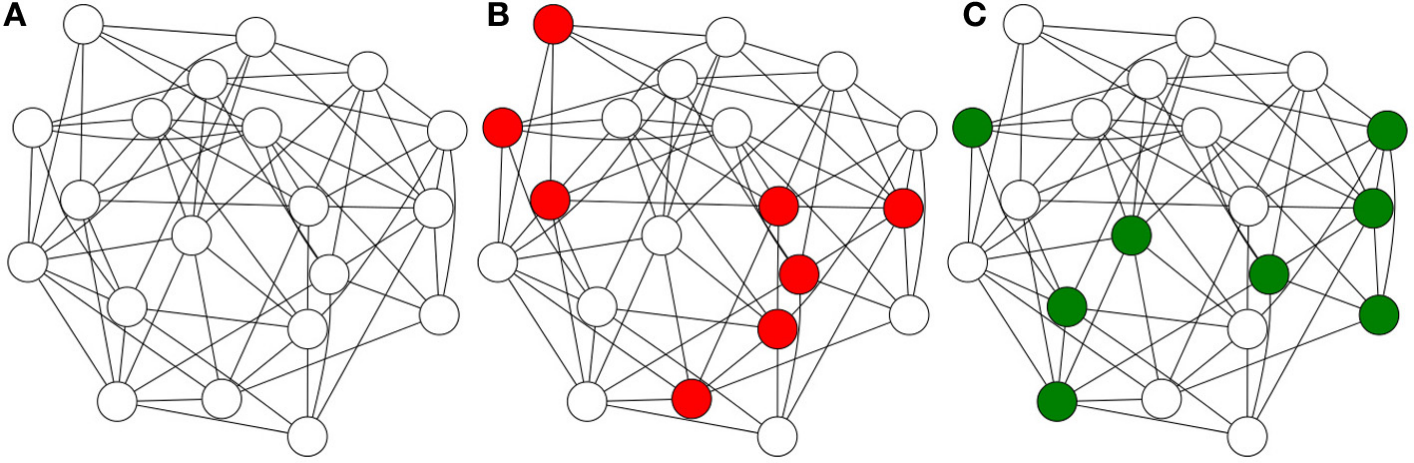
**Cell assemblies: the information encoded in a single neuron is negligible and fragile**. Multiple neurons may provide the best description of human information processing.

Hence we subscribe to the hypothesis that the unit of information encoding is not the individual neuron, but groups of neurons, or cell assemblies (Sakurai, 1996, 1998, 1999). In this model, shown in Figure 1, a single neuron may participate in a large number of assemblies, and the possible number of assemblies is enormous. Cell assemblies, rather than being encumbered by such combinatorial explosions, actually *take advantage* of them, as we will see in Sparse Distributed Memory.

### 2.2. Sparse distributed memory

A promising research programme in theoretical neuroscience is centered around *Sparse Distributed Memory*, originally proposed by Kanerva (1988). SDM is a neuroscientific and psychologically plausible model of human memory.

#### 2.2.1. A large space for memory items

SDM introduces many interesting mathematical properties of *n*-dimensional binary space that, in a memory model, are psychologically plausible. Most notable among these are robustness against noisy information, the tip-of-the-tongue phenomenon, conformity to the limits of short-term memory (Linhares et al., 2011), and robustness against loss of neurons. The model has been explored in the study of vision and other senses (Olshausen et al., 1993; Laurent, 2002; Rao et al., 2002; Mazor and Laurent, 2005). In spite of the increasing number of neuroscientists displaying interest in Sparse Distributed Memory (Ballard et al., 1997; French, 1999; Ludermir et al., 1999; Silva et al., 2004; Laurent, 2006; Bancroft et al., 2012), we still have limited understanding of its properties.

As in some other neuroscientific models, inhibitory and excitatory signals are represented in binary form. In SDM, both the data and the storage space belong to {0, 1}^*n*^, hence a particular memory item is represented by a binary vector of length *n*, henceforth called a *bitstring*. These binary bitstrings are stored (as with most computational memory models) in *addresses*. In SDM, these also take the form of *n*-dimensional binary vectors.

The distance between two bitstrings is calculated using the Hamming distance. Hamming distance is defined for two bitstrings of equal length as the number of positions in which the bits differ. For example, 00110_*b*_ and 01100_*b*_ are bitstrings of length 5 and their Hamming distance is 2.

The size of the {0, 1}^*n*^ address space grows exponentially with the number of dimensions *n*; i.e., *N* = 2^*n*^. While Kanerva (1988) suggests *n* between 100 and 10, 000, recently he has postulated 10, 000 as a desirable minimum *n* (Kanerva, 2009). This is, of course, an enormous space, unfeasible to be physically implemented.

To solve the feasibility problem of implementing this memory, SDM takes a uniformly distributed random sample of {0, 1}^*n*^, having *N*′ elements, and instantiates only these points of the space. These instantiated addresses in the sample are called *hard locations* and each hard location implements a set of *n* counters, which we will see in more detail. The hard locations allow SDM to use the entire (virtual) {0, 1}^*n*^ space through distributed read and write operations (described in more detail below). A random bitstring is generated with equal probability of 0's and 1's in each dimension. Thus, the average distance between two random bitstrings has a binomial distribution with mean μ = *n*/2 and standard deviation $\sigma =\sqrt{n/4}$. For large *n*, the vast majority of the space lies “close” to the mean (i.e., between μ − 3σ and μ + 3σ) and has few shared hard locations: as *n* grows, two bitstrings with distance far from *n*/2 are very improbable. We define two bitstrings to be *orthogonal* when their distance is close to *n*/2.

**Figure 2:**
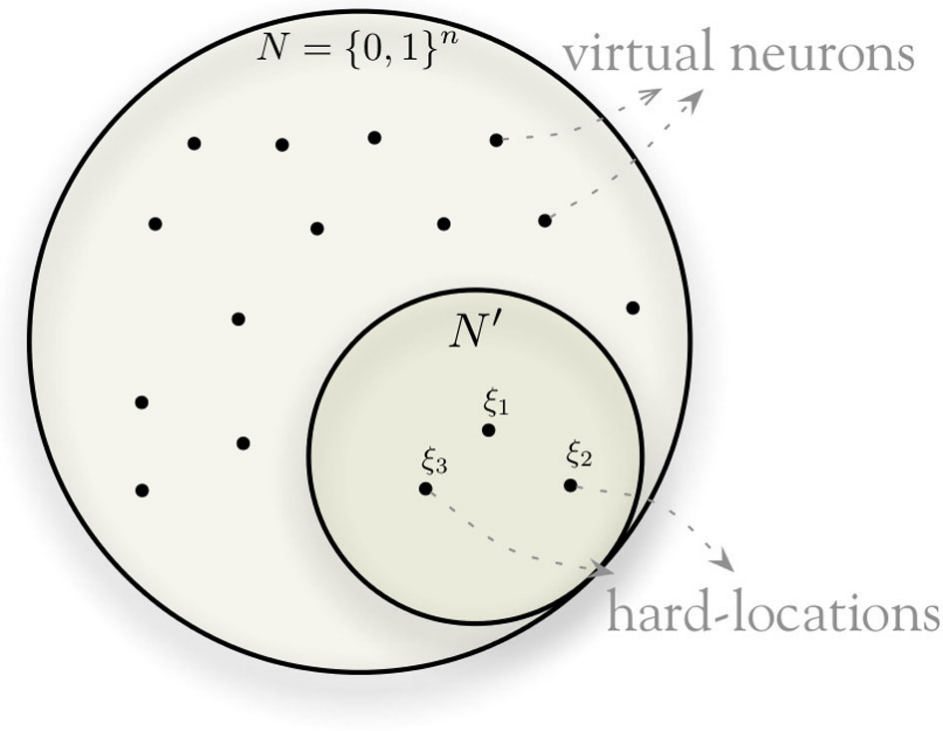
**Hard-locations randomly sampled from binary space**.

Figure 2 provides a simplified view of the model, with a small space for hard locations and a large space for possible locations. The model instantiates a random sample of about one million hard locations—which is in fact, a minute fraction of the space: for *n* = 100, only 100 · 10^6^/2^100^ = 7 · 10^−23^ percent of the whole space “exists” (i.e., is instantiated), and for *n* = 1000 only 100 · 10^6^/2^1000^ = 7 · 10^−294^ percent.

#### 2.2.2. Creating a cell assembly by sampling the space at μ − 3σ

**Figure 3:**
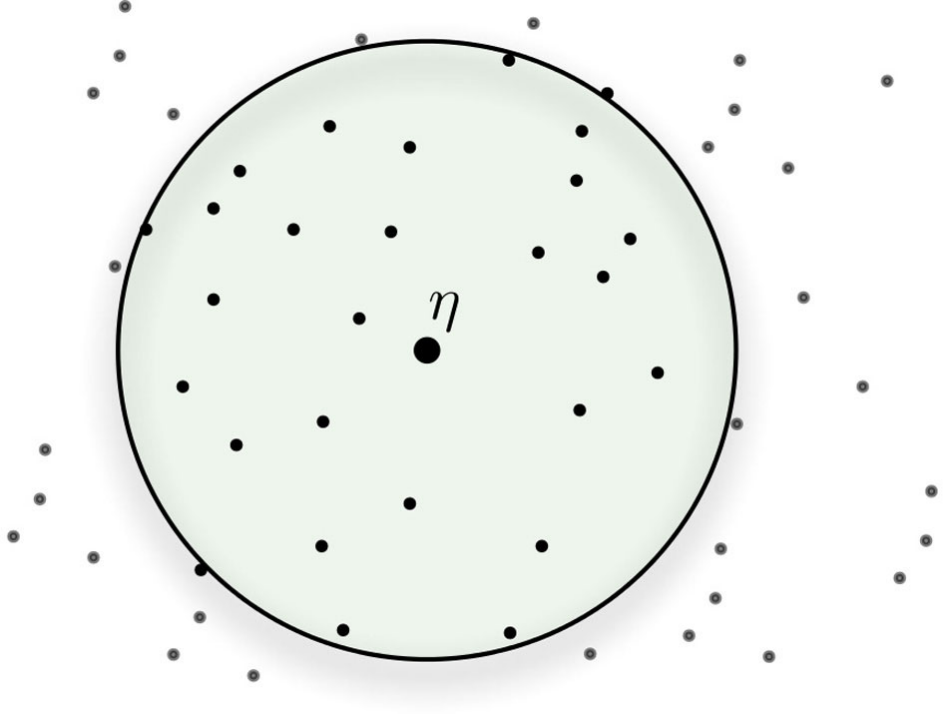
**Activated addresses inside access radius *r* around center address**.

The activation of addresses takes place according to their Hamming distance from the datum. Suppose one is writing datum η at address ξ, then all addresses inside an *n*-dimensional circle with center ξ and radius *r* are activated. So, η will be stored in *all* of these activated addresses, which are around address ξ, as shown in Figure 3. An address ξ′ is inside the circle if its hamming distance to the center ξ is less than or equal to the radius *r*, i.e., *distance*(ξ, ξ′) ≤ *r*. Generally, *r* = μ − 3σ. The radius is selected to activate, on average, 1/1000th of the sample, that is, approximately 1000 hard locations for a model with one million hard locations. To achieve this, a 1000-dimension memory uses an access radius *r* = 451, and a 256-dimensional memory, *r* = 103. This will generate a cell assembly to either store or retrieve a memory item. With this activation mechanism, SDM provides a method to write and read *any* bitstring in the {0, 1}^*n*^ space.

#### 2.2.3. Writing an item to the memory

Table 1 shows an example of a write operation being performed in a 7-dimensional memory.

**Table 1:**
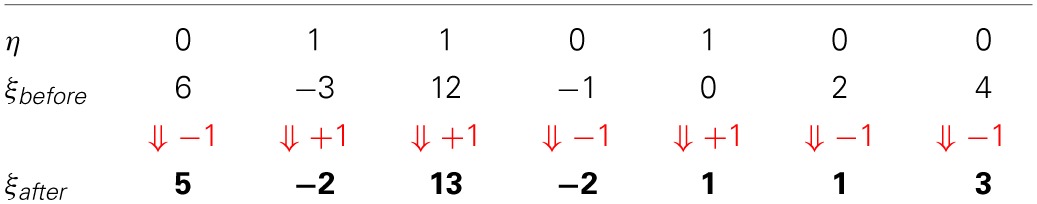
**Write operation example in a 7-dimensional memory of data η being written to ξ, one of the activated addresses**.

One way to view the write and read operations is to visualize *neurons (hard locations) as vectors*, that is vectors pointing to certain areas of the space. In the SDM model, the cell assembly (i.e., the set of active hard locations) work in unison, rather like a sum of vectors: as one writes bitstrings in memory, the counters of the hard locations are updated.

When a bitstring activates a set of hard locations, the active hard locations do not *individually* point to the bitstring that activated them, but, taken together, they point to a coordinate in space (that is, the bitstring that activated them). In this fashion, any one hard location can be said to simultaneously point to many different areas of the space, and any point in space is represented by the set of hard locations it activates.

**Figure 4:**
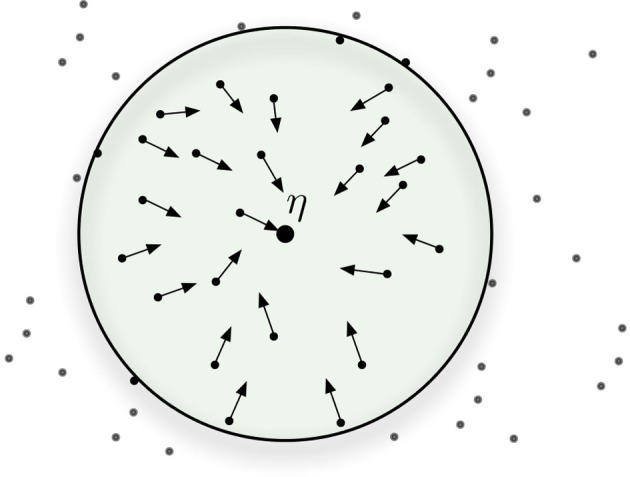
**Hard-locations pointing, approximately, to the target bitstring**.

In other words, both reading and writing depend on many hard locations to be successful. This effect is represented in Figure 4: where all hard locations inside the circle are activated and they, individually, do not point to η. But, as vectors, their sum points to the general direction of η. If another datum ν is written into the memory near η, the shared hard locations will have information from both of them and would not point (directly) to either. All hard locations, inside and outside of the circle, may also point elsewhere to other additional data points: as we have seen, even “face” neurons have multiple functions.

The write operation works as follows: Suppose one is writing datum η at address ξ: then all hard locations inside an *n*-dimensional circle with center ξ and radius *r* are activated. So, η will be stored in all these *activated* addresses, which are close to address ξ. An address ξ′ is inside the circle if its hamming distance to the center ξ is less than or equal to the radius *r*, i.e., *distance*(ξ, ξ′) ≤ *r*. The information will be written to the entire cell assembly: thus, *all hard locations within the circle* will be updated.

Each hard location has both an *address* (given by its bitstring) and a *value*. The value is stored in *counters*. Each hard location has one counter for each dimension in the space. Each counter stores, for its dimension, the bit value that has been written more frequently (0's or 1's) to its hard location. So each counter, corresponding to each dimension, is incremented for each bit 1 and decremented for each bit 0 written to that hard location. Thus, if the counter is positive, the hard location has had more 1's than 0's written to it, if the counter is negative, more 0's than 1's, and if the counter is zero, there have been an equal number of 1's and 0's written to that particular dimension in that particular hard location.

Each datum η is written into the counters of every activated hard location inside the access radius, centered on the address ξ that equals the datum: ξ = η. If some neurons are lost, only a fraction of the datum is lost, and the memory remains capable of retrieving the right datum due to the high redundancy of the model.

#### 2.2.4. Reading an item from memory

Table 2 illustrates a read operation over a 7-dimensional memory.

**Table 2:**
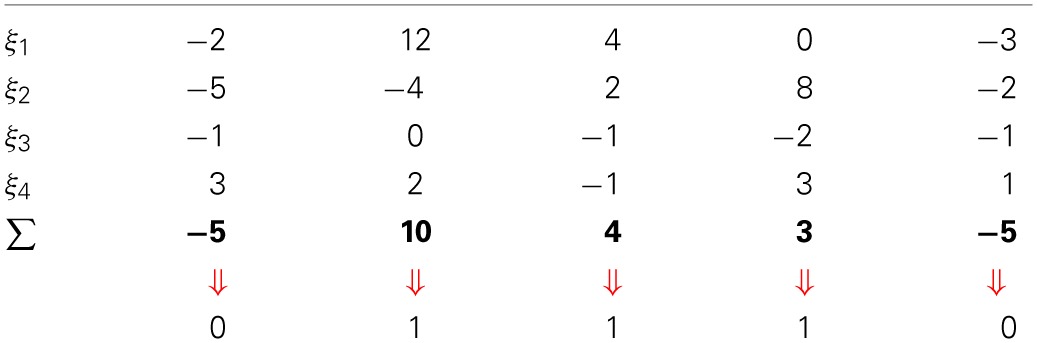
**Read operation example**.

The read operation is performed by polling each activated hard location and choosing the most-written bit for each dimension. A hard location is considered *activated* if it is within a hamming distance (radius) of the activating bitstring cue. Activated hard locations are taken into account in calculating the result of a read operation, while others are ignored. Reading consists of adding all *n* counters from the activated hard locations and, for each bit, setting it to 1 if the counter is positive, setting it to 0 if the counter if negative, and randomly setting it to 0 or 1 if the counter is zero. Thus, each bit of the returned bitstring is chosen according to all written bitstrings in the entire cell assembly (i.e., all active hard locations) and is equal to the bit value most written in that dimension. In short, the read operation depends on many hard locations to be successful. If another datum ν is written into the memory near η, the shared hard locations will have information from both of them without directly pointing to ν either. In this way, any one hard location may, in a fashion, simultaneously “point” to multiple addresses.

**Figure 5:**
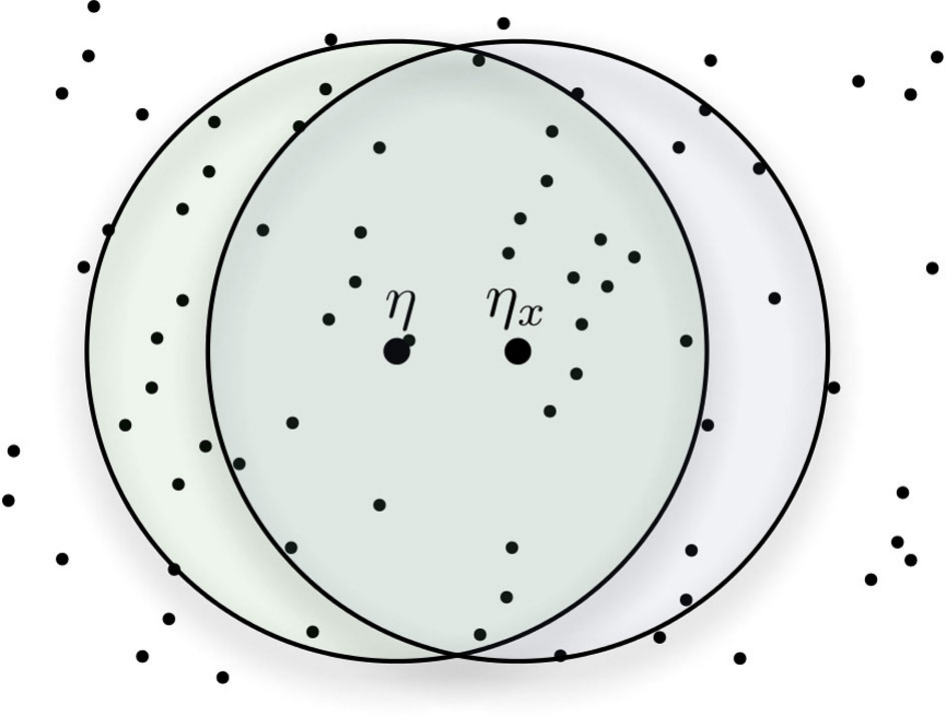
**Shared addresses between the target datum η and the cue η_*x*_**.

An imprecise cue η_*x*_ shares hard locations with the target bitstring η—yet it should be possible to retrieve η correctly, even if additional reading operations become necessary to retrieve η exactly. When reading a cue η_*x*_ that is *x* bits away from η, the cue shares many hard locations with η (see Figure 5). The number of shared hard locations decreases as the distance of the cue to η increases, in other words, as *x* = *d*(η_*x*_, η) increases. The target datum η is read in all addresses shared between η and η_*x*_, thus they will bias the read output toward the direction of η. If the cue is sufficiently close to the target datum η, the output of the read operation will be closer to η than η_*x*_ originally was. Iterating the read operation will obtain results increasingly closer to η, until it is exactly the same. So η_*x*0_ will yield an η_*x*1_ that is closer, reading at η_*x*1_ yields an η_*x*2_ that is closer still and so on until η_*xi*_ = η, if the iteration converges. Hence, performing a sequence of successive read operations will allow convergence onto the target data η.

Since a cue η_*x*_ near the target bitstring η shares many hard locations with η, SDM can retrieve data from imprecise cues (i.e., as an autoassociative memory). In spite of this characteristic, it is crucial to know how imprecise this cue could be while still converging. What is the maximum distance from our cue to the original data that still retrieves the right answer? There is a precise point in which a non-linearity occurs, and the qualitative behavior of the model changes.

A striking feature of this model is its reflection of the psychological “tip-of-tongue” phenomenon, which seems to reflect the limits of human recollection. It is the psychological state in which one knows that one knows some pre-registered memory item, yet one is unable to recollect it at a given time.

**Figure 6:**
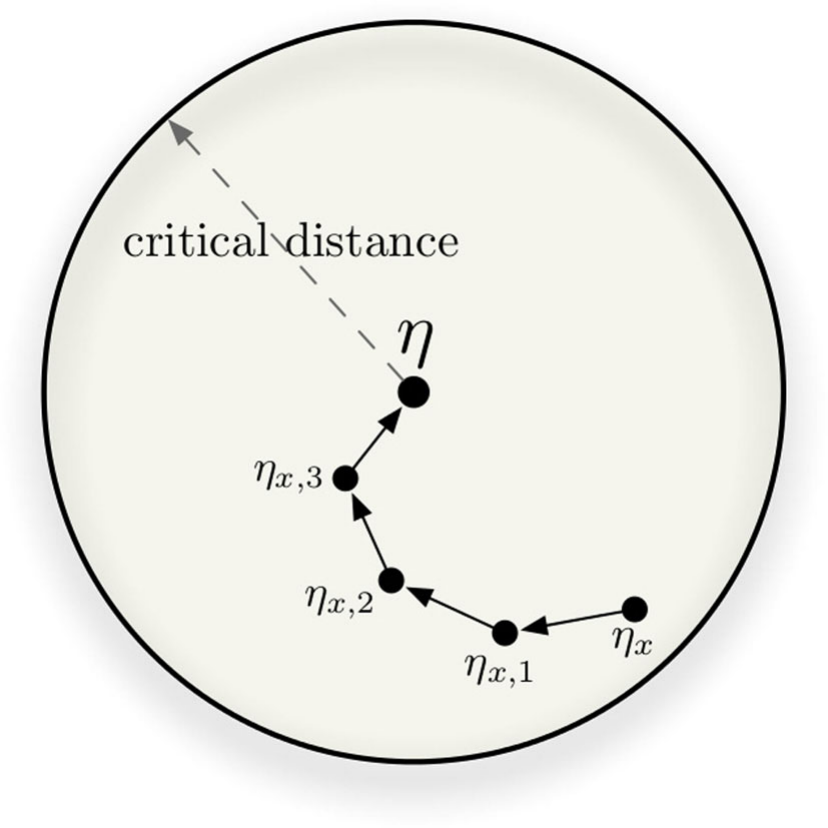
**In this example, four iterative readings are required to converge from η_*x*_ to η**.

The tip-of-the-tongue phenomenon occurs when a person knows that he/she has been previously exposed to a certain stimulus, but is unable to recall some specifics. In SDM, a tip-of-tongue memory event occurs when the expected time to convergence (or divergence) approaches infinity. In other words, when successive read iterations fail to converge or to diverge. Kanerva (1988) called this particular instance of *x*, where the output of the read operation averages *x*, the *critical distance*. Intuitively, it is the distance from which smaller distances converge and greater distances diverge. In Figure 6, the circle has radius equal to the critical distance and every η_*x*_ inside the circle should converge. The figure also shows an example of convergence in four readings. We put that this is a proxy for the edge of human recall: a threshold until which recollection occurs, and beyond which it no longer occurs.

Kanerva describes this critical distance as the threshold of convergence of a sequence of read words. It is “the distance beyond which divergence is more likely than convergence” (Kanerva, 1988). Furthermore, “a very good estimate of the critical distance can be obtained by finding the distance at which the arithmetic mean of the new distance to the target equals the old distance to the target” (Kanerva, 1988).

Kanerva has analytically derived this non-linearity for a very particular set of circumstances. His original book analyzed a specific situation with *n* = 1000 (*N* = 2^1000^), 1,000,000 hard locations, an access-radius of 451 (with 1000 hard locations in each circle) and 10,000 writes of random bitstrings in the memory. This is a very particular set of parameters, and doesn't shed light on questions of speed and robustness of expert recollection. In the next section we deal with this non-linearity and the issue of analyzing critical distance as an optimization problem.

In subsequent sections, we will derive an equation for the critical distance, in terms of SDM's parameters. We will then present empirical results of the evolution of the critical distance under varying conditions,which shed light on the model's behavior. It is worth noting that, since SDM is itself a computer simulation, what we call *empirical results* refer to conclusions obtained over data from thousands of runs of the simulation. All data and conclusions (aside from theory) herein refer to trials over computer simulations.

## 3. Materials and methods

### 3.1. Deriving the critical distance as a minimization problem

Kanerva has shown that, when 10, 000 items are stored in the memory, and the number of dimensions *N* = 1000, then the critical distance is at a Hamming distance of 209 bits: if one reads the item at a distance smaller than 209 bits, one is able to iteratively converge toward the item. If, on the other hand, one reads the item at a distance higher than 209 bits, the memory cannot retrieve the item. Furthermore at the juncture of about 209 bits, expected time to convergence grows to infinite. This reflects the aforementioned tip-of-the-tongue phenomenon: when one knows that one knows a particular bit of knowledge, yet is unable to retrieve it at that point. Psychologically, this would entail some top–down mechanism which would force the iterated search to halt. We establish a maximum number of iterated reads, based on repeated simulations (see section 4.2).

Kanerva thus fixed a number of parameters in order to derive this mathematical result:

1. the number of dimensions, *N* = 1000;
2. the number of other items stored into the memory, at 10, 000;
3. the reading method (by pooling all hard locations);
4. a single write of the target bitstring in the memory;
5. the access radius of 451, activating approximately 1000 hard locations per read or write operation.

As Kanerva defined it, approximately half of read operations 209 bits away from the target data will bring us closer to the target and approximately half will move us away from the target. His math could be simplified to this: each item will activate approximately 1000 hard locations, so writing 10, 000 items randomly will activate a total of 10, 000, 000 hard locations, giving an average of 10 different bitstrings written in each hard location. When one reads from a bitstring η_200_, 200 bits away of the target η, η_200_ will share a mean of 97 hard locations with the target (Kanerva, 1988, Table 7.1, p. 63). This way, it is possible to split the set of active hard locations into two groups: one group having 903 hard locations with 10 random bitstrings written into each; and other group having 97 hard locations each with 9 random bitstrings plus our target bitstring η.

Let us analyze what happens to each bit of the read bitstrings. To each bit we have 903 · 10 + 97 · 9 = 9903 random bits out of a total of 10, 000 bits. The total number of 1-bits is a random variable that follows the Binomial distribution with 9903 samples and *p* = 0.5. It has a mean of 9903/2 = 4951.5 and standard deviation $\sqrt{9903/4}=49.75$. If our target bit is 0 we will choose correctly when our sum is less than half total, or 10, 000/2 = 5000. If our target bit is 1, our sum is the random variable of total 1-bits added by 97 1-bits from our sample. Adding a constant number changes only the mean and does not affect the standard deviation. So we will choose correctly when our sum of means 4951.5 + 97 = 5048.5 and standard deviation 49.75 is greater than 5000. Both probabilities here equal 83% of choosing the same bit as the target. As we have 1000 bits, in average, we can predict that the result of the read operation will be 170 bits away from the target.

The critical distance is the point where the aforementioned probability equals the distance from the bitstring η_*x*_ to the target η, or *x* = *n*(1 − *p*), where *x* is the distance from the bitstring to the target, *p* is the probability of choosing the wrong value of a bit (given by the above technique), and *n* is the number of dimensions.

Given that we intend to study the critical distance as a theoretical proxy for the limits of human recollection, we would like to explore a larger number of possibilities and parameter settings of the model. Hence we compute the non-linearity of the critical distance as minimization problem. Let:

- *d*: be the distance to the target;
- *h*: be the number of hard locations activated during read and write operations (note that this value depends on that access radius);
- *s*: be the number of total stored bitstrings in the SDM;
- *H*: be the number of instantiated hard locations;
- *w*: be the number of times the target bitstring was written in memory;
- θ: be the total of random bitstrings in all *h* hard locations activated by a read operation; i.e., the size of a cell assembly; and
- ϕ(*d*): be the mean number of shared hard locations activated by two bitstrings *d* bits away from each other. One can find values for a 1000-dimensional SDM in Kanerva's book, Table 7.1, p. 63, or the equations to calculate to any SDM in (de PáduaBraga and Aleksander, 1995; Kanerva, 1988, Appendix B, p. 125).

Consider a memory in which a total of *s* bitstrings have already been stored via write operations. Each of these write operations would have activated approximately *h* hard locations. This way, on average, all write operations together activate a total of *sh* hard locations. This gives an average of *sh*/*H* random bitstrings stored in each hard location.

Knowing the average number of bitstrings stored in each hard location, it is simple to find an equation for θ. Each read operation performed for a cue η_*d*_ has ϕ(*d*) hard locations shared with the target bitstring η, and *h* − ϕ(*d*) non-shared hard locations. The non-shared hard locations have only random bitstrings stored in themselves. However, the shared hard locations have the target bitstring written *w* times, resulting in fewer random bitstrings. As the average number of bitstrings written in each hard locations is *sh*/*H*, we have:

$$\begin{array}{l} \theta =\frac{s\cdot h}{H}\cdot \left[h-\phi (d)\right]+\left(\frac{s\cdot h}{H}-w\right)\cdot \phi (d)\\ \theta =\frac{s\cdot h^{2}}{H}-w\cdot \phi (d) \end{array}$$

Suppose the *k*-th bit of our target bitstring is zero. The read operation will correctly choose bit 0 if, and only if, more than half of the bitstrings from the activated hard locations has the *k*-th bit 0 (setting aside the case of an equal number of zeros and ones^1^). As each hard location has *sh*/*H* bitstrings and the read operation activates *h*, half of the bitstrings equals *h* · *sh*/(2*H*) = *sh*^2^/(2*H*). Then, to choose correctly, we should have $\sum_{i=1}^{\theta }X_{i}<sh^{2}/(2H)$, where *X*_*i*_ is the *k*-th bit of the *i*-th bitstring stored in each activated hard location.

Suppose the *k*-th bit our target bitstring is 1. The read operation will choose bit 1 when more than half of the bitstrings from the activated hard locations has the *k*-th bit 1. We have already seen that half of the bitstrings is *sh*^2^/(2*H*). But here, as the bit equals 1 and there are *w* target bitstrings in each ϕ(*d*), we have to add *w* · ϕ(*d*) to the sum. In other words, we must account for the number of times the target was written into the hard locations which are activated by both the target and the cue which is at a distance *d*. This gives us $w\cdot \phi (d)+\sum_{i\;=\;1}^{\theta }X_{i}>sh^{2}/(2H)$.

Summarizing, we have:

$$\begin{array}{l} P(wrong|bit=0)=1-P\left(\sum_{i=1}^{\theta }X_{i}<\frac{sh^{2}}{2H}\right)\\ P(wrong|bit=1)=P\left(\sum_{i=1}^{\theta }X_{i}<\frac{sh^{2}}{2H}-w\cdot \phi (d)\right) \end{array}$$

We already know that *P*(*X*_*i*_ = 1) = *P*(*X*_*i*_ = 0) = 1/2. Since each *X*_*i*_ corresponds to a Bernoulli trial, $\sum_{i\;=\;1}^{\theta }X_{i}\sim Binomial(\theta ;0.5)$, which has mean θ/2 and standard deviation $\sqrt{\theta /4}$.

The critical distance is the distance where the chance of convergence to the target equals the distance of divergence from the target. That is, in the critical distance, the probability of a wrong choice of the bit, times the number of bits, is equal to the original distance to the target. Then, the critical distance is the *d* that satisfies equation *P*(*wrong*) · *n* = *d* or *P*(*wrong*) = *d/n*.

Using the theorem of total probability, we have:

$$\begin{array}{l} P(wrong)=P(wrong|bit=0)\cdot P(bit=0)\\ \;+P(wrong|bit=1)\cdot P(bit=1) \end{array}$$

if we let

$$\alpha =P\left(\sum_{i=1}^{\theta (d)}X_{i}<\frac{sh^{2}}{2H}\right)$$

and

$$\beta =P\left(\sum_{i=1}^{\theta (d)}X_{i}<\frac{sh^{2}}{2H}-w\cdot \phi (d)\right)$$

thus,

$$P(wrong)=\frac{1}{2}\cdot \left[\left(1-\alpha \right)+\beta \right]$$

This way, the equation to be solved is:

$$\frac{1}{2}\cdot \left[\left(1-\alpha \right)+\beta \right]=\frac{d}{n}$$

Since *d* is an integer value and θ is a function of *d*, this equality may not be achievable (this describes a range, where for a certain *d*: *left side* > *right side* and for *d* + 1: *left side* < *right side*). In these cases, the critical distance can be obtained minimizing the following equation with the restriction of *d* ∈ ℕ and *d* ≤ *n*:

$$f(d)={\left\{\frac{1}{2}\cdot \left[\left(1-\alpha \right)+\beta \right]-\frac{d}{n}\right\}}^{2}$$

If the size of the cell assembly, θ, is large enough, a good approximation to the *Binomial*(θ; 0.5) is the normal distribution. Let *N* be the normalized normal distribution with mean zero and variance one. We have:

$$\begin{array}{l} \alpha ≃N\left(z<\frac{sh^{2}/(2H)-\theta /2}{\sqrt{\theta /4}}\right)=\tilde{\alpha }\\ \beta ≃N\left(z<\frac{sh^{2}/(2H)-w\cdot \phi (d)-\theta /2}{\sqrt{\theta /4}}\right)=\tilde{\beta } \end{array}$$

Simplifying, we have:

$$\begin{array}{l} \;N\left(z<\frac{sh^{2}/(2H)-\theta /2}{\sqrt{\theta /4}}\right)=N\left(z<\frac{w\cdot \phi (d)}{\sqrt{\theta }}\right)\\ N\left(z<\frac{sh^{2}/(2H)-w\cdot \phi (d)-\theta /2}{\sqrt{\theta /4}}\right)=N\left(z<\frac{-w\cdot \phi (d)}{\sqrt{\theta }}\right) \end{array}$$

And we have to minimize the following function with restrictions of *d* ∈ ℕ and *d* ≤ *n*:

$$\tilde{f}(d)={\left\{\frac{1}{2}\cdot \left[1-\tilde{\alpha }+\tilde{\beta }\right]-\frac{d}{n}\right\}}^{2}$$

In the case studied in Kanerva (1988), *n* = 1000, *h* = 1000, *H* = 1, 000, 000, *s* = 10, 000, *w* = 1, and θ = 10, 000 − ϕ(*d*). Replacing these values in the equation, we have to minimize:

$$\tilde{f}(d)={\left\{\frac{1}{2}\cdot \left[1-\tilde{\alpha }+\tilde{\beta }\right]-\frac{d}{1000}\right\}}^{2}$$

When *d* = 209, we have ϕ(*d*) = 87 and $\tilde{f}$(209) ≅ 0.00032, which is the global minimum.

We note once again that equations to calculate ϕ(*d*) have been derived in Kanerva (1988, Appendix B) and need not be repeated here—see also the derivations for higher *d* by de Pádua Braga and Aleksander (1995). The example calculated above used Table 7.1 of Kanerva (1988), which has the values of ϕ(*d*) for *d* in a 1000-dimensional SDM with one million hard locations.

In the following section we briefly reiterate and discuss the contribution of the theoretical model, and turn to empirical results pertaining to the exploration of the critical distance. We vary parameters of the memory model in order to explore the changes to the critical distance. These empirical trials yield enlightening results pertaining to the critical distance as a parallel for the edge of human recollection and for human expertise in SDM.

## 4. Results

In this text, we show that, given $\tilde{\alpha }$, $\tilde{\beta }$, and *d*, minimizing the function (repeated here from the previous derivation):

$$\tilde{f}(d)={\left\{\frac{1}{2}\cdot \left[1-\tilde{\alpha }+\tilde{\beta }\right]-\frac{d}{n}\right\}}^{2}$$

solves the issue of non-linearity involved in the critical distance of the model, that is, the psychological limits of human recollection at a given point in time. Such result should be valuable to assess whether the memory is prone to convergence or divergence.

This result may help provide avenues of exploration in theoretical neuroscience and can be readily available to cognitive modelers. Yet, it still falls short of giving us an intuitive understanding of the speed and robustness of the memory of experts. Therefore, we will explore the critical distance behavior at different configurations. We have implemented the model and conducted a large set of computational experiments, whose visualizations illuminate the issue of expert memory.

### 4.1. Numerical simulations: visualizing the memory dynamics

So far we have seen a single particular case with set parameters, and our goal is to understand the speed and robustness of expert memory. Let us consider variations of these parameters, and compute, through simulations, the behavior of the critical distance. We vary the number of dimensions *N* ∈ {256, 1000}, we vary the number of stored items from the set {1000, 2000, …, 50000}, and we vary the rehearsal number: the number of times an item has been stored in the memory.

The following figures depict heat maps describing the behavior of the critical distance. In these simulations, all items are stored at their respective locations, that is, a bitstring *x* is stored at the location *x*. Generating each heat map proved computationally demanding: when *N* = 1000, approximately 305, 000, 000, 000, 000 bit-compares are required (storage of items in memory: 5 · 10^13^, and to read items from memory: 3 · 10^15^ bit-compares). *Each individual pixel* demands an average of 7, 000, 000, 000 bit-compares.

All figures presented below have three colored lines. The green line marks the *first occurrence of non-convergence* to the exact target bitstring. The red line marks the *last occurrence of the convergence* to the exact target bitstring. Finally, the blue line marks the *estimated critical distance*, that is where the read output, on average, equals the input distance to the target bitstring. It is an estimation because the critical distance is not exactly defined this way. Critical distance is the point or region in which both divergence and convergence have a 50% chance to occur. That is, all points before the green line converged, all points after the red line diverged, and the points between these lines sometimes converge and sometimes diverge.

One may notice that, despite not having an exact convergence, almost all points between the green and the red line are near the target bitstring.

### 4.2. Influence of iterative readings in critical distance

The number of iterative-readings is an important parameter of an SDM implementation. Simulations were done in a 1000 and 256-dimensional SDM. Both with one million hard locations, activating (on average) 1000 hard locations per operation and varying the number of times the target bitstring η is written to memory.

For each write-strength of η (written once, twice, five times, nine times) we varied the saturation of the SDM, that is, the number of random bitstrings written (once each) along with *eta* in the memory. We varied this from 1000 to 50, 000 random bitstrings, in increments of 1000. Once populated with *eta* plus the random bitstrings, we performed 1–40 iterative-readings at each possible distance from the target (from zero to the number of dimensions).

**Figure 7:**
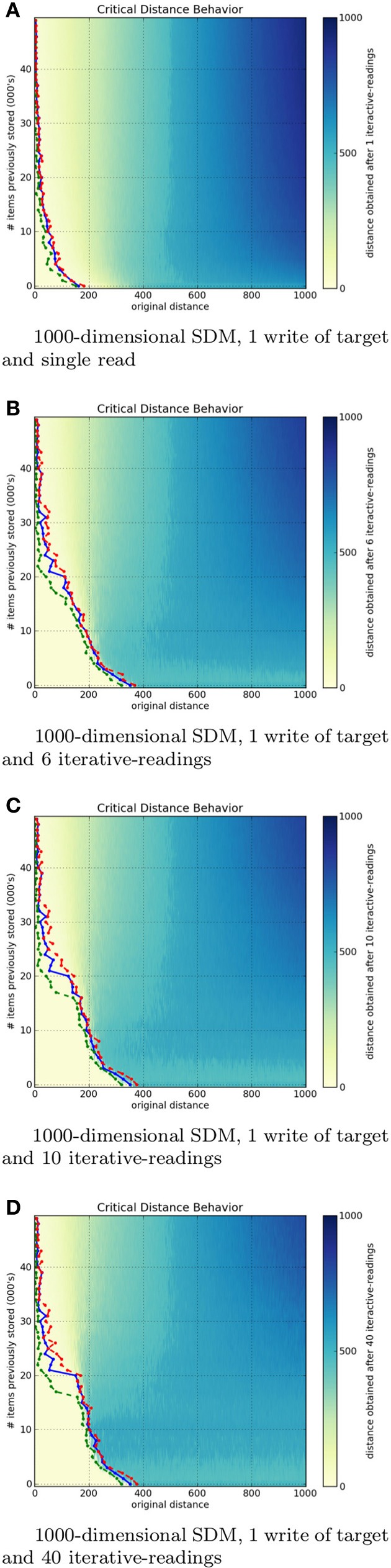
**Influence of number of iterative-readings in a 1000-dimen- sional SDM memory**.

Figures 7A–D show, respectively, a 1000-dimensional SDM checked with a single read, 6, 10, and 40 iterative-readings. It is easy to see a huge difference from a single read to more reads, but a small difference from 6 to 10 and from 10 to 40 iterative-readings. These observations also apply to our tests with the 256-dimensional SDM. As compared to the 1000-dimensional SDM, we found a smaller, more gradual difference from a single read to more reads, yet a minute difference from 6 to 10 and from 10 to 40 iterative-readings. Following these results, due to the number of computations needed in each simulation, all other simulations were done using 6 iterative-readings, since 40 iterative-readings have only a slight improvement in relation to six.

It is unexpected that, after 40, 000 writes in the 1000-dimensional memory, the critical distance is so small. Kanerva (1988) showed that, under these parameters, the memory capacity is slightly less than 100, 000 items. The author defines SDM capacity as saturated when its critical distance is zero. In the 256-dimensional memory, this behavior starts after 20, 000 writes. This is unexpected, since Kanerva's estimation for *N* = 256 is between 112, 000 and 137, 000 random bitstrings stored.

Our principal hypothesis for the discrepancies between our empirical results and the original theory is that, while the hard locations are instantiated as samples from a uniform distribution and our simulations wrote bitstrings randomly, they do not saturate uniformly. Any write activates a fixed average (around 1000 in our case) of hard locations, but the variance in this case is not insignificant. One bitstring read may activate 900 while another (in another area of the space, be it close or far) may activate 1100 hard locations. Thus, certain hard locations would become more noise than signal during activation sooner, rather than a uniform degradation occurring. This discrepancy would cause, in the aggregate, a saturation of the SDM with fewer bitstrings stored than expected in theory. This remains one possibility, though we hope the issue will be explored in future work.

### 4.3. Influence of the number of writes on the critical distance

The influence of the number of writes on the critical distance was not analyzed by Kanerva. It is important because, when a random bitstring is seen only once, it is psychologically plausible that it will be gradually forgotten with new incoming information. What matters is not exactly the number of writes, but the proportion of the number of times a bitstring was stored in relation to others.

A remark on cognitive psychology is in order here. Consider, as an example, the aforementioned exchange between Szilard and Einstein. As an expert confronts unexpected information, it is reasonable to expect that additional memory writes will occur. If we presume that evolution brought the human memory close to optimality, as explored by the rational analysis approach (Anderson and Milson, 1989; Anderson, 1990; Anderson and Schooler, 1991), one would expect some mechanism akin to Shannon's idea of *information content* to be in play.

That is, as an expert is surprised by new, unforeseen, information, say, an outcome Θ, with information *I*(Θ) = −*log*(*P*(Θ)), where *I* stands for the information content in outcome Θ. One would therefore expect the expert's memory to either place additional attention to the outcome, leading to: (1) additional writes to memory, or (2) amplification of the write operation's signals, or possibly (3) both effects.

**Figure 8:**
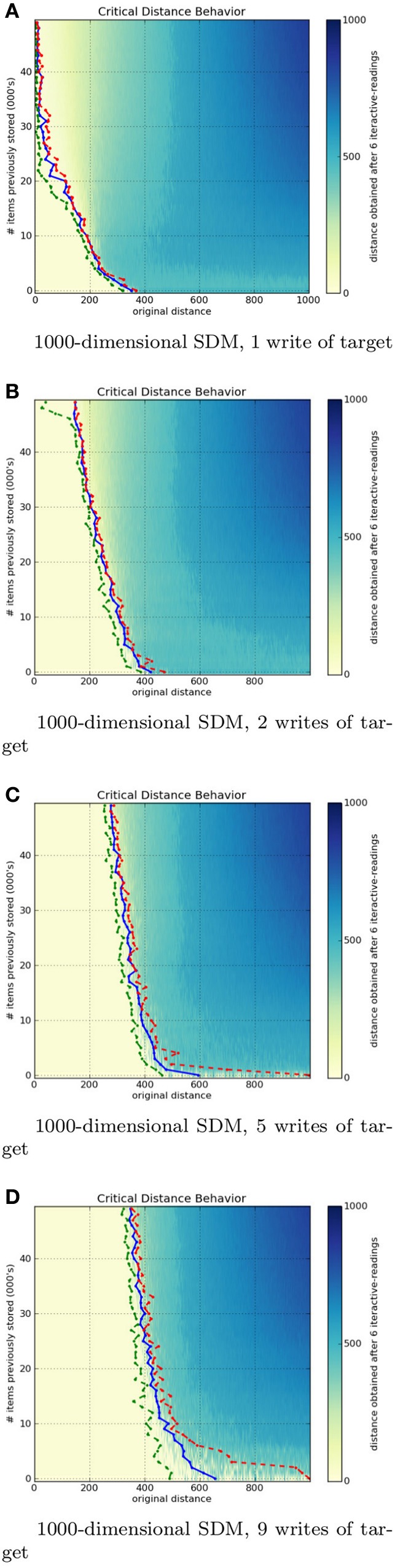
**Influence of number of target writes in a 1000-dimensional SDM memory**.

Figures 8A–D show a 1000-dimensional SDM with 1, 2, 5, and 9 writes of the target bitstring η. It is easy to see a huge difference from 1 to 2 writes. Although the green line has a strange behavior near 50, 000 items stored, the critical distance was much greater than with 1 write. From 2 to 5 to 9 rehearsals, the critical distance starts growing rapidly and slows down near six writes. This makes sense, since it should have a threshold smaller than 500 bits.

The 256-dimensional memory has a similar behavior, but less abrupt. It keeps growing, but slower than a 100-dimensional memory. It never crosses the 50 bits on x-axis in 256 bits, while the 1000-dimensional reaches the 200 bits on x-axis and almost hits 400 bits on the x-axis.

These figures display the immense power of reinforcement or rehearsal: additional writes of a memory item significantly raise the attractor basin (critical distance) for that memory item.

This behavior is plausible, as the human brain rapidly recognizes a pattern when it is used to it. Many times, the patterns appear in different contexts, giving cues far from the target concept, much like a chess player, who looks at a position and rapidly recognizes what is happening (Bilalić et al., 2009; Rennig et al., 2013).

## 5. Discussion

This is the first work focused on better understanding the critical distance behavior of a Sparse Distributed Memory (Kanerva, personal communication). Our future research intends to explore the rehearsal mechanisms in cognitive architectures for one of the most studied domains of expertise: (Linhares, 2005; Linhares and Brum, 2007; Linhares and Freitas, 2010; Linhares et al., 2012; Linhares and Chada, 2013; Linhares, 2014), and attempt to bridge the low-level world of neurons and their assemblies with the high-level world of abstract thought and understanding of strategic scenarios. We have argued here that, as SDM remains both a psychologically plausible and a neuroscientifically plausible model of human memory, the study of its critical distance may provide insights into the edges of our own recollection. Without a precise understanding of the critical distance behavior, one cannot advance the theoretical model. Moreover, one cannot develop robust applications without knowing the limits of convergence.

The empirical tests shown here confirmed that the critical distance in SDM constitutes a “band” wherein both convergence and divergence become less and less likely. This is a palatable result because, intuitively, the Tip-of-the-Tongue phenomenon in humans seems like an attractor, something we sometimes “fall into.” We argue this is a parallel between SDM and human recollection, and posit that our theoretical and empirical results provide evidence that the critical distance is a correlate to the edge of human recollection.

While humans sometimes fall into the TotT, there are also times when we *almost* fall into it and, after a bit of effort, are able to recall the desired information. In the model, this would mean we enter the critical band, but leave it after one or two iterations and converge. Likewise, it seems one can be very certain of what one is saying and, in mid-sentence, completely diverge from the next piece of information we wished to recall. In SDM this would amount to entering the critical band, but then diverging.

As Figure 6 shows, the speed of convergence is a function of the number of read operations: additional read operations bring one closer to the memory item (assuming that the original cue was not past the critical point). We also see that this effect is greatly reduced after 6 to 10 read operations. As Figure 7 shows, expertise can be correlated with providing additional writes to the memory, and we show that increasing the rehearsal number *greatly increases the margin for error or ambiguity*, and *greatly decreases the relevant information needed for convergence*, as the critical threshold is increased. In human terms, experts “know what you are talking about” with fewer cues. Their memory has much greater robustness.

Yet, it is *the combination of these two dynamics* that sheds light on experts' speed. Taking the SDM model as a plausible account of human memory, we can compare by saying that, for experts, having a much higher threshold may signify *being able to converge within fewer, or even a single, read operation*. As the hard locations have been reinforced with the original information, read operations converge faster. With very few cues and noisy, ambiguous, information, experts may still manage to recollect and understand almost immediately the object, situation, or event in question. It is no wonder Albert Einstein could immediately grasp Leo Szilard's concerns.

### 5.1. Data sharing

All the computational methods developed in this study are available as an open-source project, and can be found at https://github.com/msbrogli/sdm.

## Funding

This work has been generously supported by grants from the Fulbright Foundation, and FAPERJ Foundation (grants E-26/110.540/2012 and E-26/111.846/2011), grants from the CNPq Foundation (grants 401883/2011-6 and 470341/2009-2), the Pro-Pesquisa program of FGV Foundation.

### Conflict of interest statement

The authors declare that the research was conducted in the absence of any commercial or financial relationships that could be construed as a potential conflict of interest.

## Footnotes

^1^the case for a random coin toss is negligible, since, as θ becomes large, its probability tends toward 0 quickly.

## Supplemental material

The supplemental data file consists of five parts: (1) An introduction to the computational methods available in https://github.com/msbrogli/sdm; (2) A large set of additional heatmaps, documenting the behavior of the model in a 1000-dimensional memory; (3) The same tests on 256-dimensional memory, (4) all rehearsal results for 256-dimensional memory, and (5) the same tests for 1000-dimensional memory.
